# Antimicrobial Action of Water-Soluble β-Chitosan against Clinical Multi-Drug Resistant Bacteria

**DOI:** 10.3390/ijms16047995

**Published:** 2015-04-10

**Authors:** Seong-Cheol Park, Joung-Pyo Nam, Jun-Ho Kim, Young-Min Kim, Jae-Woon Nah, Mi-Kyeong Jang

**Affiliations:** Department of Polymer Science and Engineering, Sunchon National University, 255 Jungang-ro, Suncheon, Jeonnam 540-950, Korea; E-Mails: schpark9@gnu.ac.kr (S.-C.P.); always_shh@naver.com (J.-P.N.); mmoi@naver.com (J.-H.K.); metingym@sunchon.ac.kr (Y.-M.K.)

**Keywords:** β-chitosan, multidrug-resistant bacteria, antibacterial activity, low molecular water-soluble chitosan

## Abstract

Recently, the number of patients infected by drug-resistant pathogenic microbes has increased remarkably worldwide, and a number of studies have reported new antibiotics from natural sources. Among them, chitosan, with a high molecular weight and α-conformation, exhibits potent antimicrobial activity, but useful applications as an antibiotic are limited by its cytotoxicity and insolubility at physiological pH. In the present study, the antibacterial activity of low molecular weight water-soluble (LMWS) α-chitosan (α1k, α5k, and α10k with molecular masses of 1, 5, and 10 kDa, respectively) and β-chitosan (β1k, β5k, and β10k) was compared using a range of pathogenic bacteria containing drug-resistant bacteria isolated from patients at different pH. Interestingly, β5k and β10k exhibited potent antibacterial activity, even at pH 7.4, whereas only α10k was effective at pH 7.4. The active target of β-chitosan is the bacterial membrane, where the leakage of calcein is induced in artificial PE/PG vesicles, bacterial mimetic membrane. Moreover, scanning electron microscopy showed that they caused significant morphological changes on the bacterial surfaces. An *in vivo* study utilizing a bacteria-infected mouse model found that LMWS β-chitosan could be used as a candidate in anti-infective or wound healing therapeutic applications.

## 1. Introduction

The emergence of multidrug-resistant (MDR) pathogens has become an increasingly a serious problem in the field of antibiotic therapeutics [1,2]. Therefore, new antibiotics, such as phytochemical and synthetic chemical materials [3,4], antimicrobial peptides [2,5], and inhibitors for drug-efflux pumps [6,7,8], have attracted attention for overcoming MDR pathogens. Among them, this study proposes low molecular water-soluble (LMWS) β-conformational chitosan to protect MDR bacteria.

Chitosan is polysaccharide that is composed of β-(1-4)-linked d-glucosamine and *N*-acetyl-d-glucosamine. The material is normally prepared from chitin, which has been found in a wide range of natural sources (crustaceans, fungi, insects, annelids, mollusks, coelenterate, *etc.*) [9]. Chitin has three crystalline forms, α-, β-, and γ-chitins. The α-chitin forms an anti-parallel orientation with inter-chain and intra-sheet hydrogen bonds in the adjacent chains [10]. The β-chitin adopts a parallel orientation that forms intra-sheet hydrogen bonds [11]. The structure of γ-chitin was proposed to be an arrangement of two parallel and one antiparallel sheets [12]. Chitosan from α-chitin is used mainly in various biological and biomedical fields because of their abundance and easy accessibility [13]. β-chitin might be a promising alternative source of chitin with distinctive features due to the weak intermolecular interaction, and chitosan derived from β-chitin has strong potential as a functional biopolymer.

Recently, the applications of chitosan to medicine, food, chemical engineering, pharmaceuticals, nutrition, environmental protection and agriculture have attracted considerable interest [14,15,16,17,18]. In particular, the antifungal and antibacterial activity of α-conformational chitosan (α-chitosan) has been reported in a number of studies [17,19,20]. On the other hand, there are few biological and biotechnological applications of β-conformational chitosan (β-chitosan). The antibacterial activity of β-chitosan-based films has been reported [21,22]. In addition, the antibacterial activity between α- and β-chitosan was compared according to molecular weights ranging from 31 to 76 kDa and the degree of deacetylation (DDA), resulting in 75% DDA/31 kDa β-chitosan to 75% DDA/31 kDa α-chitosan, whereas 90% DDA/74–76 kDa α-chitosan had a lower minimum inhibitory concentration (MIC) than 90% DDA/74–76 kDa β-chitosan against *Escherichia coli* [20].

This study compared the antibacterial activity of low molecular weight water-soluble (LMWS) α- and β-chitosan with 1, 5, and 10 kDa molecular weight at pH 5.4 and 7.4 against a range of drug-susceptible bacteria. Moreover, the MICs of LMWS β-chitosan were evaluated against drug-resistant *Pseudomonas aeruginosa* and *Staphylococcus aureus*, which were isolated clinically from patients. The mode of action of LMWS β-chitosan was suggested using biophysical methods and the *in vivo* antibacterial activity of β-chitosan was evaluated using the drug-resistant bacteria-infection mouse model.

## 2. Results and Discussion

Chitin is an abundant and useful biopolymer in organisms, such as shrimp, crab, squid pens, fungi and yeast. The material is composed structurally of α, β, and γ forms. The β-chitin in this study has an affinity for solvents and is more reactive because it is constructed to a parallel structure and has no inter-hydrogen bonds. In contrast, α-chitin forms an anti-parallel structure with inter and intra-hydrogen bonds [23,24,25]. This study estimated that β-chitosan will exhibit potent antibacterial action when β-chitin is deacetylated and re-hydrated in aqueous solution due to structural differences between two-forms of chitin.

### 2.1. A Potent Antibacterial Activity of LMWS β-Chitosan

The antibacterial activities of LMWS α-chitosan and β-chitosan series were examined against 10 strains of drug-susceptible bacteria in two pH values (pH 5.4 and 7.4). As shown in Table 1, α10k and β10k chitosan with a molecular mass of 10 kDa inhibited the growth of all tested bacteria at 0.005~0.018 mg/mL. The α5k chitosan exhibited strong antibacterial action in only pH 5.4, whereas β5k chitosan had low MICs of 0.009~0.018 mg/mL in pH 5.4 and 7.4. On the other hand, α1k chitosan had a more active effect than β1k chitosan at pH 5.4; the MIC against *E. coli* O157 and *S. epidermidis* was 0.009 mg/mL.

**Table 1:** Antibacterial activity of water-soluble α- and β-chitosan against drug-susceptible bacteria. Antimicrobial assays were performed in 10 mM sodium phosphate (pH 5.4 and 7.4) containing 10% culture medium.

Bacteria	Minimum Inhibitory Concentration (MIC, mg/mL)											
	α1k		α5k		α10k		β1k		β5k		β10k	
pH	7.4	5.4	7.4	5.4	7.4	5.4	7.4	5.4	7.4	5.4	7.4	5.4
**Gram(−) bacteria**												
*E. coli*	>5	0.156	>5	0.039	0.009	0.009	0.625	1.25	0.018	0.009	0.009	0.009
*E. coli O157*	>5	0.009	>5	0.009	0.009	0.009	0.625	1.25	0.009	0.009	0.009	0.009
*P. aeruginosa*	>5	0.156	>5	0.078	0.009	0.009	0.625	1.25	0.009	0.009	0.009	0.009
*S. typhimurium*	>5	0.045	>5	0.009	0.009	0.005	0.313	1.25	0.018	0.009	0.009	0.009
*L. monocytogens*	>5	0.313	>5	0.156	0.009	0.009	0.313	2.5	0.009	0.009	0.009	0.009
**Gram(+) bacteria**												
*B. cereus*	>5	0.018	>5	0.018	4.5	0.009	0.625	0.625	0.009	0.009	0.009	0.009
*B. megaterium*	2500	0.018	313	0.018	0.009	0.009	0.313	1.25	0.009	0.009	0.009	0.009
*S. aureus*	>5	0.039	>5	0.018	0.009	0.009	1.25	2.5	0.018	0.009	0.009	0.009
*S. epidermidis*	5	0.009	5	0.045	0.018	0.005	0.625	2.5	0.018	0.009	0.018	0.005

This pattern was similar to the drug-resistant *P. aeruginosa* strains, but β-chitosan was better against the drug-resistant *S. auresus* strains (data not shown). The antibacterial activity of β5k and β10k chitosan was more effective against the drug-resistant *P. aeruginosa* and *S. aureus* strains, which were isolated clinically from patients, than against the drug-susceptible strains or conventional antibiotics (Table 2). Mostly, β-chitosan was better than α-chitosan in the inhibition of bacterial growth. Generally, the antibacterial activity of chitosan is affected by an interaction between the positively charged chitosan (protonated amine groups) and the negatively charged bacterial membrane (lipopolysaccharide in Gram-negative bacteria and lipoteichoic acid in Gram-positive bacteria) [17]. In addition, the hydrophobicity of the glucopyranoside rings of chitosan contributes to the insertion or translocation into the hydrophobic tail part of lipid. Therefore, the potent antibacterial activity of β-chitosan is due to structural organization because it is flexible, allowing an increase in the contacting regions with the bacterial surfaces in an aqueous solution, whereas α-chitosan is rigid because of the strong intra- and inter-hydrogen bonds. Moreover, the free amine groups of β-chitosan may be protonated easily but α-chitosan will have low protonated amine groups because of the inter-hydrogen bonds in the inter-amines. 

**Table 2:** Antibacterial activity of water-soluble β-chitosan and antibiotics against drug-resistant bacteria. Antibacterial assay was performed in pH 7.4.

Bacteria	MIC (μg/mL)						
	β5k	β10k	PIP ^a^	AMP ^a^	CIP ^a^	GM ^a^	TET ^a^
**Gram(−) bacteria**							
*P. aeruginosa* ATCC 27853	9	9	<4	<2	0.25	0.5	–
*P. aeruginosa* BMP-Pa001	<4.5	<4.5	>128	>32	2	1	–
*P. aeruginosa* BMP-Pa002	<4.5	<4.5	16	>32	>8	8	–
*P. aeruginosa* BMP-Pa003	<4.5	<4.5	>128	>32	>8	32	–
*P. aeruginosa* BMP-Pa004	<4.5	<4.5	16	>32	0.25	1	–
*P. aeruginosa* BMP-Pa005	<4.5	<4.5	>128	>32	0.25	8	–
**Gram(+) bacteria**							
*S. aureus* ATCC 25923	18	9	–	–	0.25	0.25	0.5
*S. aureus* BMP-Sa001	<4.5	<4.5	–	–	1	0.25	>16
*S. aureus* BMP-Sa002	39	18	–	–	>8	>16	1
*S. aureus* BMP-Sa003	39	18	–	–	>8	>16	>16
*S. aureus* BMP-Sa004	18	9	–	–	>8	>16	>16
*S. aureus* BMP-Sa005	39	18	–	–	>8	>16	>16

*P. aeruginosa* BMP-Pa and *S. aureus* BMP-Pa strains are resistant-strains isolated from patients in the hospital. ^a^ PIP, AMS, CIP, GM, and TET are piperacillin, ampicillin, ciprofloxacin, gentamicin, and tetracycline, respectively.

### 2.2. Cytotoxic Effect of Chitosan

**Figure 1:**
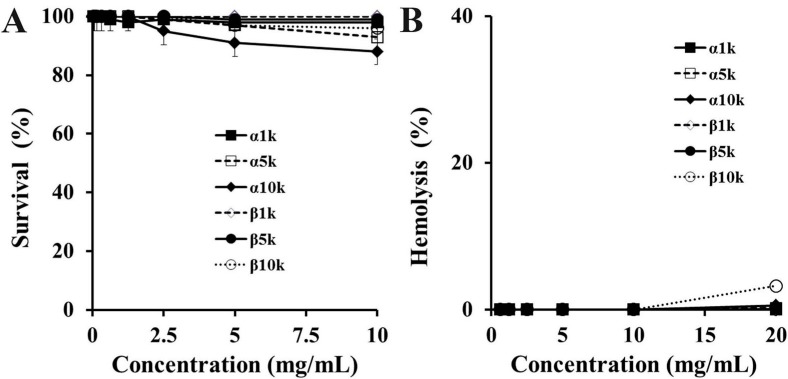
Cytotoxic and hemolytic effects of α-chitosan and β-chitosan samples against HaCaT cells (**A**) and rRBC (**B**), respectively. (**A**) HaCaT cells were exposed for 24 h to different concentrations of samples and percentage of cell survival was determined by the MTT (3-(4,5-dimethylthiazol-2-yl)-2,5diphenyltetrazolium bromide) assay; (**B**) Dose-dependent hemoglobin release activity was measured after 1 h of incubation with rat erythrocytes (8% hematocrit). All graphs represent the mean ± S.E.M. of values obtained from at least two independent experiments performed in triplicate.

To assess the cytotoxicity of chitosan, hemolysis and a MTT assay were performed against human erythrocytes and HEK 293 cells, respectively. None of the chitosan forms showed significant lytic activity at 10 mg/mL (Figure 1). The *in vivo* safety of chitosan is very important. In particular, the therapeutic effect of β-chitosan was distinguishable because a cytotoxic assay was performed and its antibacterial activity was effective in the physiological pH (~pH 7.4), which suggests that it is a promising candidate for the development of antibiotic agents.

### 2.3. Membranolytic Action of β-Chitosan

**Figure 2:**
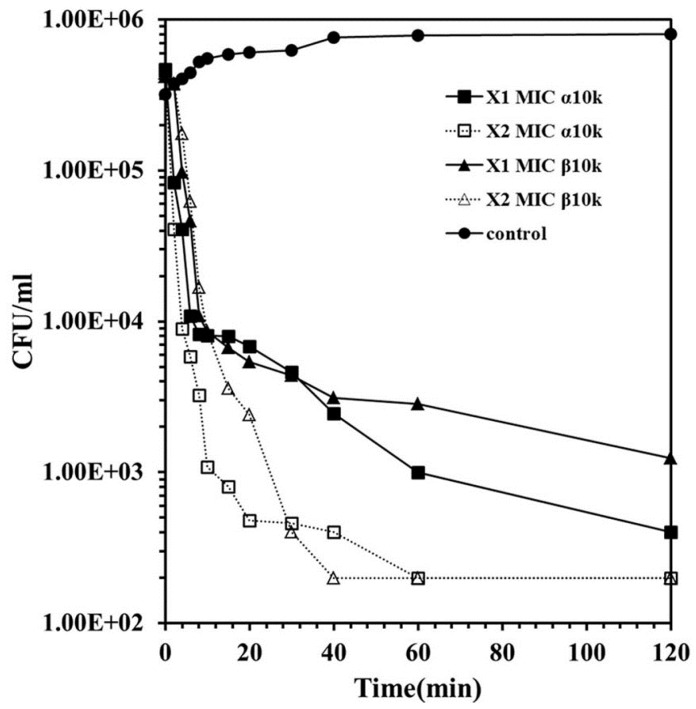
Time-kill kinetic of α-chitosan and β-chitosan samples in *E. coli* O157. Bacterial cells were exposed to samples at one or two times concentration of their minimum inhibitory concentration (MIC) values in pH 7.4 and incubated for 0–120 min.

**Figure 3:**
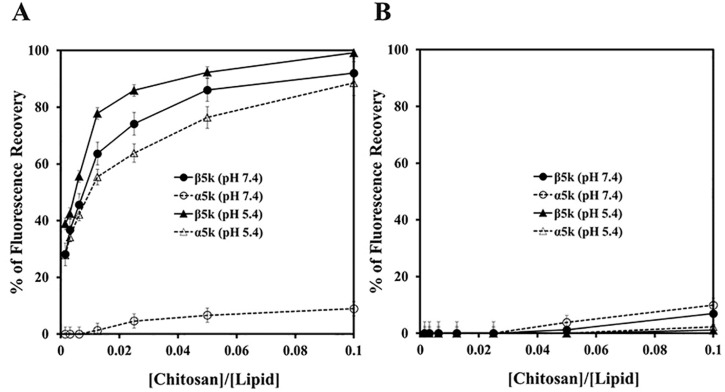
Rhodamine leakage in l-α-phosphatidylethanolamine (PE)/l-α-phosphatidylglycerol (PG) (**A**) and l-α-phosphatidylcholine (PC)/cholesterol (CH)/sphingomyelin (SM) (**B**) vesicles.

Although the overall antibacterial mechanism of chitosan has not been elucidated clearly, inhibition of the synthesis of DNA or a disruption of the bacterial cell membrane was suggested [17]. Figure 2 shows the time-kill kinetics of α10k and β10k chitosan against *E. coli* O157 at one or two times the MIC. The kill kinetics of β10k chitosan was similar to α10k chitosan, and both forms showed bacteriocidal activity. A significant decrease in the bacterial colonies within 10 min indicates the membranolytic action of chitosan. To examine the membrane-permeabilizing action of chitosan precisely, artificial liposome (PE/PG, 7:3, *w*/*w*), a mimetic bacterial membrane, was used by entrapping fluorescent dye, rhodamine. As shown in Figure 3A, β5k chitosan induced significant dye-leakage at pH 5.4 and 7.4 in a dose-dependent manner, whereas the fluorescence intensity in α5k chitosan was very low at pH 7.4, showing similar antibacterial activity. α10k and β10k chitosan showed high fluorescent intensity in pH 5.4 and 7.4 (data not shown). The dye-leakage effects of them were low in a mammalian mimetic membrane (PC/Ch/SM, 2:1:1, *w*/*w*/*w*), despite the high weight ratio (Figure 3B).

**Figure 4:**
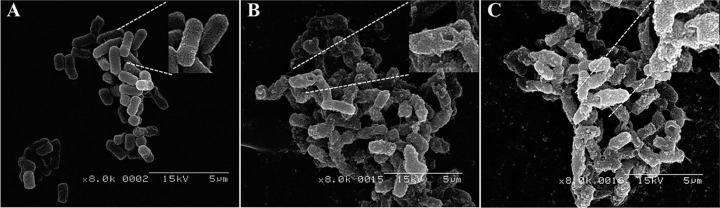
Morphological alteration of *E. coli* cells in the absence (**A**) or presence of β5k (**B**) or β10k (**C**).

The morphological changes induced by β5k and β10k chitosan were examined by SEM. The untreated *E. coli* O157 had a normal smooth surface (Figure 4A), whereas cells exposed to β-chitosan showed cell surface disruption and small blebs (Figure 4B,C). These results suggest that the mode of action of β-chitosan is membranolytic.

### 2.4. In Vivo Antibacterial Effect of β-Chitosan

**Figure 5:**
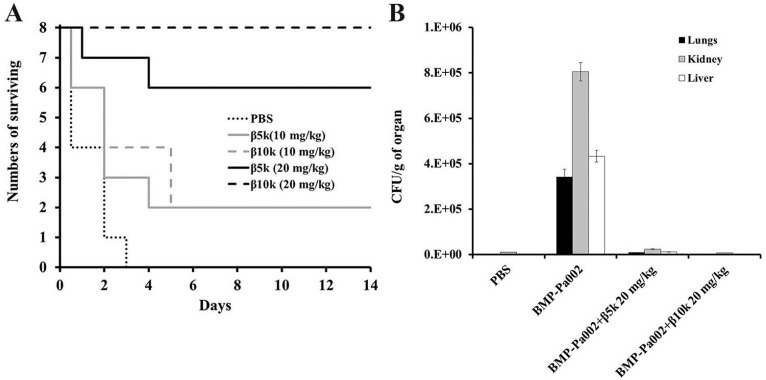
Evaluation of β5k and β10k against *P. aeruginosa* BMP-Pa002 in a bacteria-infected mouse model. (**A**) Survival rates of ICR (Institute for Cancer Research) mouse with β-chitosan. ICR mouse were infected by intraperitoneally injection with *P. aeruginosa* BMP-Pa002. After 1 h, samples were injected at the indicated concentrations by single dose; (**B**) After mice treated with each samples were sacrificed, lung, kidney, and liver were homogenized. Viable bacteria were counted on an agar plate.

**Figure 6:**
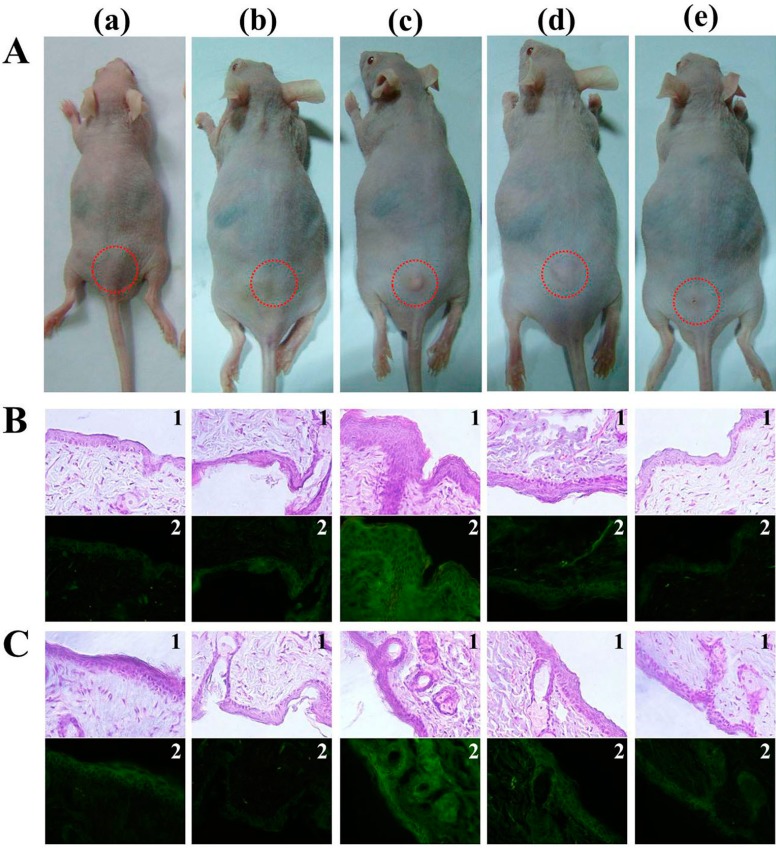
Topical application of beta-chitosan on the back skin of hairless mouse causes histological changes of dermatitis. *S. aureus* BMP-Sa003 (5 × 10^8^ CFU/mL) was injected into back skin of hairless mouse, following injection of 0.5 or 1 mg/mL of β10k chitosan. After immolation of mice (**A**) at 7 days, the tissues of dorsal skin was stained with hematoxylin and eosin staining (H & E) (**1**), and detected TNF-α (**B**) and IL-1β (**C**) with FITC (Fluorecein isothiocyanate)-labeled (**2**) secondary antibody. The sectioned tissues were observed of magnification 40× under the inverted microscopy. (**a**) PBS (Phosphate-buffered saline); (**b**) 1 mg/mL of β10k; (**c**) bacteria; (**d**) bacteria and 0.5 mg/mL of β10k; (**e**) bacteria and 1 mg/mL of β10k.

The *in vivo* antibacterial action of β5k and β10k chitosan was analyzed in ICR mice infected with drug-resistant *P. aeruginosa* BMP-Pa002 (*n* = 8). After administering 10 and 20 mg/kg of β-chitosan by an intraperitoneal injection, the mice were monitored for 14 days. The results revealed a good survival rate of mice treated with β10k (20 mg/kg) (Figure 5A). In addition, the bacterial colonization showed that the mice in the presence of β-chitosan was reduced significantly (Figure 5B). To apply the topical treatment of β-chitosan, *S. aureus* BMP-Sa003 was injected into the skin epidermal layer of the ICR mice (Figure 6). After 7 days, the mice were sacrificed and the dorsal skin was stained with hematoxylin and eosin staining (H & E). Although serious damage, such as the tissue construction and infiltration of mast cells, was observed in the untreated mice (negative control), the skin tissue of the mice in the presence of β-chitosan had recovered to a similar level to that of the control. Furthermore, the induced inflammation in the hairless mouse skin was determined by measuring the pro-inflammatory cytokine (TNF-α and IL-1β). The H & E stained sections showed that the epidermal thickness was increased by *S. aureus* BMP-Sa003 cells, whereas the skin inoculated by only 1 mg/mL of β-chitosan was unchanged (Figure 6A). After a bacterial infection, a treatment with β-chitosan showed a repaired epidermis thickness (Figure 6A). The cytological characteristics were examined by immunohistochemistry (IHC) using TNF-α (Figure 6B) and IL-1β (Figure 6C) antibodies. The fluorescence of the skin sections showed a significant increase in the *S. aureus* inoculated groups, compared to the untreated and only β-chitosan. The pro-inflammatory cytokines of the treated groups was reduced in a dose-dependent manner (Figure 6B,C).

## 3. Experimental Section

### 3.1. Materials

Chitosan oligosaccharide (α-chitosan from crab shell and β-chitosan from squid pen, 98% deacetylated chitosans, average molecular weight of 9 kDa) was provided from Chittolife Co., Ltd. (Seoul, Korea). Carboxytetramethylrhodamine succinimidyl ester (TAMRA), ampicillin, piperacillin, ciprofloxacin, gentamicin, and tetracycline were purchased from Sigma Chemical Co. (St. Louis, MO, USA). l-α-phosphatidylethanolamine (PE, from *E. coli*), sphingomyelin (SM, from porcine brain), cholesterol (Ch, from ovine wool), l-α-phosphatidylglycerol (PG, from *E. coli*) and l-α-phosphatidylcholine (PC, from chicken egg) were obtained from Avanti Polar Lipids (Alabaster, AL, USA). All other reagents were of analytical grade. The buffers were prepared in double-glass-distilled water.

### 3.2. Preparation of Low Molecular Weight Water-Soluble Chitosans (LMWSC)

Five grams of chitosan oligosaccharide (α- and β-chitosan) were dissolved in 10 mL of phosphate-buffered saline (pH 7.0). In order to remove the lactic acid salt attached to the C-2 position of the glucose amine units, approximately 5.2 mL of triethylamine (TEA) were then slowly added to the chitosan solution, which was then stirred for 2 h at room temperature. Subsequently, acetone was added to precipitate the chitosan, and the mixture was then centrifuged (12,000 rpm, 20 min, 4 °C). The resulting product was treated with aliquots of 0.001 N HCl for an additional 2 h and then washed five-times. The product was then combined and air dried. To remove the residual HCl and other impurities, the product was dissolved in double distilled water and dialyzed by pure distilled water. The resulting aqueous solution was then lyophilized [26]. The LMWSCs were separated by ultrafiltration through cut-off membranes. LMWSCs were divided into α1k, α5k, and α-10k as α-chitosan, and β-1k, β5k, and β10k as β-chitosan according to the molecular weight determined by gel permeation chromatography (GPC).

### 3.3. Bacterial Strains

*Listeria monocytogenes* (ATCC 19115), *Staphylococcus aureus* (ATCC 25923), *Pseudomonas aeruginosa* (ATCC 27853), *Escherichia coli* (ATCC 25922), and *E. coli* O-157 (ATCC 43895) were purchased from the American Type Culture Collection (ATCC). *Streptococcus epidermidis* (KCTC 3096), and *Salmonella typhimurium* (KCTC 1926), *Bacillus megaterium* (KCTC 3007) were obtained from the Korean Collection for Type Cultures (KCTC) in Korea Research Institute of Bioscience & Biotechnology (KRIBB) (Taejon, Korea). *P. aeruginosa* BMP-Pa001~005 and *S. aureus* BMP-Pa001~005 were resistant strains isolated from the patients in a hospital.

### 3.4. Antibacterial Activity

A microdilution assay was conducted to establish the minimum inhibitory concentration (MIC) values of the samples. Briefly, the bacteria were collected during their mid-log phase and suspended in 10 mM sodium phosphate (pH 5.4 or 7.4) supplemented with 10% culture media. Aliquots of the cell suspension (5 × 10^5^ colony forming unit (CFU)/mL) were added to each well with two-fold serial dilutions of each sample, followed by incubation at 37 °C for 24 h. The MICs of the samples were obtained by measuring the turbidity of each well at the absorbance at 600 nm and optical microscopy observations.

### 3.5. Kinetics of Bacterial Killing

The kinetics of bacterial killing of chitosan was evaluated using *E. coli* O-157. Mid-log phase bacteria (2 × 10^6^ CFU/mL) were incubated with 2 × MIC in a SP buffer (pH 5.4 or 7.4) containing 10% (*v*/*v*) growth medium. Aliquots were removed at fixed time intervals, diluted appropriately, plated on an agar plate, and the CFU was then counted after 24 h incubation at 37 °C.

### 3.6. Hemolysis

The hemolytic activities of the samples were assessed using rat RBCs that had been collected using heparin. The fresh rRBCs were then rinsed three times in phosphate-buffered saline (PBS) by 10 min of centrifugation at 800× *g*, after which they were resuspended in PBS. The chitosan dissolved in PBS was then added to 100 μL of the stock rRBCs suspended in PBS (final RBC concentration, 8% *v*/*v*). The samples were then incubated with agitation for 60 min at 37 °C, and then centrifuged for 10 min at 800× *g*. The absorbance of the supernatants was then assessed at 414 nm. In addition, the controls for zero hemolysis (blank) and 100% hemolysis, which consisted of rRBCs suspended in PBS and 1% Triton X-100, respectively, were evaluated. All measurements were carried out in triplicate.

### 3.7. Rhodamine Release from Liposomes

The rhodamine-entrapped liposomes were prepared for use in the dye leakage experiments as follows. Rhodamine-entrapped LUVs (large unilamellar vesicles) were prepared by vortexing the dried lipid in a dye buffer solution (10 mM rhodamine, 10 mM HEPES, 0.1 mM EDTA, pH 5.4 and pH 7.4). The suspension was then freeze-thawed in liquid nitrogen for nine cycles. The calcein-entrapped vesicles were then separated from free calcein by gel filtration chromatography on a Sephadex G-50 column. The LUVs were filtered through a polycarbonate filter (0.2-μm pore size filter) using an Avanti Mini-Extruder (Avanti Polar Lipids Inc., Alabaster, AL, USA). The entrapped LUVs in the suspensions containing 20 μM lipids were incubated with various concentrations of chitosan, which were diluted with 10 mM HEPES (pH 5.4 or 7.4). Subsequently, the fluorescent intensity of the released calcein was measured using a spectrofluorometer (Perkin-Elmer LS55, Perkin-Elmer, Waltham, MA, USA) at an excitation and emission wavelength of 480 and 520 nm, respectively. The control for complete dye leakage was determined in Triton-X 100. 100% dye leakage was achieved by the addition of Triton X-100 to a final concentration of 0.1%. The percentage leakage was then plotted. All experiments were carried out at 25 °C, and the apparent percentage of calcein release was calculated using the following equation:

Release (%) = 100 × (*F* − *F*_0_)/(*F*_t_ − *F*_0_) (1)

where *F* and *F*_t_ represent the fluorescence intensity before and after the addition of the detergent, respectively, and *F*_0_ represents the fluorescence of the intact vesicles.

### 3.8. Morphological Observation by Scanning Electron Microscopy

The morphological alterations of the *E. coli* O157 cells in the presence or absence of β-chitosan were observed by scanning electron microscope. Pre-grown *E. coli* O157 was diluted to 10^8^ CFU/mL in sodium phosphate buffer (pH 7.4), and incubated with β1k or β10k at 37 °C. The control was run in the absence of chitosan. After 30 min, the cells were fixed with 5% (*v*/*v*) glutaraldehyde in 0.2 M sodium-cacodylate buffer (pH 7.4). After 2 h of fixation at 4 °C, the samples were filtered on isopore filters (0.2 μm pore size, Millipore, Bedford, MA, USA), and washed extensively with 0.1 M cacodylate buffer (pH 7.4). The filters were then treated with 1% (*w*/*v*) OsO_4_, washed in 5% (*w*/*v*) sucrose in cacodylate buffer, and dehydrated subsequently with a graded series of ethanol. After critical point drying and gold coating, the samples were observed by scanning electron microscopy (JSM-7100F, JEOL, Tokyo, Japan)

### 3.9. In Vivo Study

*In vivo* studies were performed in accordance with the National Institutes of Health guidelines for the ethical treatment of animals, and all animal studies were approved by the Animal Care Committee of Sunchon National University (SCNU_IACUC-2013-7, 1 July 2013).

Five-week-old ICR mice were injected intraperitoneally with multidrug-resistant *P. aeruginosa* BMP-Pa002 (5 × 10^7^ CFU/mL), followed by an intraperitoneal injection of β5k and β10k chitosan (10 or 20 mg/kg) or PBS (negative control). The survival rate was recorded daily over a 14-days period. Mice with each treatment were sacrificed, and the lungs, kidneys, and liver were removed. The organs were homogenized in PBS and diluted in ten times. Subsequently, 100 μL of the diluted samples was spread on Mueller–Hinton agar. After culturing overnight, the number of observed colonies was counted.

The back skin of hairless mice was injected with drug-resistant *S. aureus* BMP-Sa003 (5 × 10^8^ cell/mL), followed by an injection of chitosan 1 day later. At 7 days, the mice skin was extracted, and washed once with phosphate buffered saline (PBS), transferred to 4% paraformaldehyde for 24 h and dehydrated 3 times with 50% to 100% ethanol for 2 h. Subsequently, xylene substituted for 1 h, and the paraffin embedded samples were cut to 4 μm (Microtome, Thermo-scientific, Waltham, MA, USA). Each sample was incubated for 30 min at room temperature with the primary antibody anti-TNF-α or anti-IL-1β in 5% (*w*/*v*) bovine serum albumin (BSA). The samples were washed with the buffer and incubated with FITC-labeled secondary antibodies, after which were stained with hematoxylin and eosin staining (H & E), and examined by fluorescence microscopy (IX71, Olympus, Tokyo, Japan).

## 4. Conclusions

Chitosan is a natural polymer derived from chitin that is used widely as a dietary supplement and in a variety of pharmacological and biomedical applications. In particular, this study evaluated the antibacterial activity of LMWS β-chitosan against drug-susceptible and drug-resistant bacterial strains. The results showed that the mode of action of β-chitosan was membranolytic, it had potent antibacterial activity against drug-resistant bacteria *in vitro* and *in vivo*, and was non-cytotoxic. Therefore, β-chitosan has potential use as a specific pharmacological agent. 
